# Virus Evolution and Neutralization Sensitivity in an HIV-1 Subtype B′ Infected Plasma Donor with Broadly Neutralizing Activity

**DOI:** 10.3390/vaccines9040311

**Published:** 2021-03-25

**Authors:** Yuanyuan Hu, Sen Zou, Zheng Wang, Ying Liu, Li Ren, Yanling Hao, Shasha Sun, Xintao Hu, Yuhua Ruan, Liying Ma, Yiming Shao, Kunxue Hong

**Affiliations:** State Key Laboratory of Infectious Disease Prevention and Control, National Center for AIDS/STD Control and Prevention, Chinese Center for Disease Control and Prevention, Beijing 102206, China; yyh5223@163.com (Y.H.); zousen@hotmail.com (S.Z.); wangzheng@chinaaids.cn (Z.W.); yingliu@chinaaids.cn (Y.L.); li.ren@chinaaids.cn (L.R.); haoyl@chinaaids.cn (Y.H.); sha837982611@hotmail.com (S.S.); xth38302@gmail.com (X.H.); ruanyuhua92@chinaaids.cn (Y.R.); mal@chinaaids.cn (L.M.); yshao@bjmu.edu.cn (Y.S.)

**Keywords:** HIV-1, envelope gene, recombination, broadly neutralizing activity, neutralizing antibodies

## Abstract

We sought to analyze the evolutionary characteristics and neutralization sensitivity of viruses in a human immunodeficiency virus type 1 (HIV-1) subtype B′ infected plasma donor with broadly neutralizing activity, which may provide information for new broadly neutralizing antibodies (bNAbs) isolation and immunogen design. A total of 83 full-length envelope genes were obtained by single-genome amplification (SGA) from the patient’s plasma at three consecutive time points (2005, 2006, and 2008) spanning four years. In addition, 28 Env-pseudotyped viruses were constructed and their neutralization sensitivity to autologous plasma and several representative bNAbs were measured. Phylogenetic analysis showed that these env sequences formed two evolutionary clusters (Cluster I and II). Cluster I viruses vanished in 2006 and then appeared as recombinants two years later. In Cluster II viruses, the V1 length and N-glycosylation sites increased over the four years of the study period. Most viruses were sensitive to concurrent and subsequent autologous plasma, and to bNAbs, including 10E8, PGT121, VRC01, and 12A21, but all viruses were resistant to PGT135. Overall, 90% of Cluster I viruses were resistant to 2G12, while 94% of Cluster II viruses were sensitive to 2G12. We confirmed that HIV-1 continued to evolve even in the presence of bNAbs, and two virus clusters in this donor adopted different escape mechanisms under the same humoral immune pressure.

## 1. Introduction

Despite nearly 40 years of effort, human immunodeficiency virus type 1 (HIV-1) infection is still a major health threat around the globe. Due to the rapid replication dynamic, error-prone reverse transcriptase, and simultaneous lack of proofreading function, latent infection, and high recombination rate, HIV-1 possesses high genetic variability and the tendency for new variants to emerge [1,2]. Among various mechanisms, recombination is an evolutionary “shortcut”. It can create a new combination of existing genetic polymorphisms in a single replication cycle, allowing the virus to acquire a broader phenotype and thus have more opportunities to escape immune stress [3].

The discovery of broadly neutralizing antibodies (bNAbs) brings new hope to design effective prophylactic vaccines and develop treatment strategies. First, bNAbs target relatively conserved regions on the envelope trimer spike and are effective against a wide range of viral isolates. Some bNAbs can neutralize more than 90% of circulating viral isolates tested [4,5]. Second, bNAbs developed during the course of HIV-1 infection [6,7,8], which provide a prototype of the bNAbs that should be elicited by a vaccine. Third, bNAbs can protect against Simian/Human Immunodefiency Virus (SHIV) challenge in animal experiments [9,10] and control virus rebound in clinical trials [11,12]. Therefore, they provide the promise of helping to guide vaccine and therapeutic strategy development.

bNAbs evolve from early neutralizing antibodies (NAbs). At the early stage of infection, NAbs have been considered ineffective because they do not seem to be associated with the control of viral replication and disease progression [13,14]. However, NAbs exert selective pressure on the viral population, leading to continuous viral variation, especially the envelope (env) region, which is the sole target of NAbs. Viral variation may induce a heterologous neutralizing response and stimulate the development of new NAb specificities, further broadening the broadly neutralizing activity in chronically infected individuals [15], and a limited percentage of these infectors are able to develop bNAbs [16]. However, despite decades of research and different kinds of immunogen designs, bNAbs have not yet been elicited by vaccination [17,18].

Understanding viral characteristics related to the development of bNAbs in natural HIV-1 infection may offer valuable information for successful vaccine design. Early coevolving relationships between viruses and autologous neutralizing antibodies have been dissected in several studies [19,20,21]. However, little is known about the characteristics of viruses in chronic HIV-1 infectors who develop bNAbs or the mechanism of virus escape in the presence of these bNAbs. This knowledge will be helpful for designing an efficient vaccine and help to determine which kinds of bNAbs may be contained in the sample, which provides a basis for the separation and identification of new bNAbs in later stages.

Here, we focus on a chronic HIV-1 infector with a broadly neutralizing activity that provides us with a unique opportunity to study the virus’s evolutionary characteristics under bNAb pressure. From this patient’s plasma, 83 full-length HIV-1 env gene sequences were amplified longitudinally at three time points (2005, 2006, and 2008) spanning four years. These env sequences clustered and formed two evolutionary clusters (Cluster I and II) on the phylogenetic tree. The two clusters evolved in different ways under the same immune pressure in an individual. Cluster I recombined, and Cluster II increased the length of the V1 ring and glycosylation to escape immune pressure. Then, 28 representative env clones were expressed on pseudoviruses. We tested the viral neutralization sensitivities to autologous plasmas and several well-known bNAbs to analyze the immune pressure from bNAbs.

## 2. Materials and Methods

### 2.1. Profile of an HIV-1 Subtype B′ Chronically Infected Donor

The slow progressor CBJC515 described in this study was infected with HIV-1 subtype B’ through unregulated commercial plasma donation between 1992 and 1995 [22]. The blood samples for this study were firstly collected on 16 August 2005 and then collected on 18 April 2006 and on 18 November 2008. The patient’s viral load increased from 19,700 to 125,000 copies/mL, and his CD4 T cell count decreased from 528 to 321 cells/µL during the study period, indicating that his disease progressed over time (Table 1). The patient was referred to receive antiviral treatment in 2009 due to a low CD4 T cell count of less than 350 cells/µL.

CBJC515 was identified to possess broadly neutralizing activity, the neutralization breadth and the geometric mean ID50 titer of his plasma were 95.65% and 256.57, respectively, when tested against DRVI(Division of Research on Virology and Immunology) panel of 23 pseudoviruses, including eight clade B strains (two tier 3 strains and six tier 2 strains), four clade A strains of tier 2, four C strains (three tier 2 and one tier 1B), four CRF07_BC strains (three tier 2 strains and one tier 3 strain) and three clade CRF01_AE strains (Table 2). The neutralizing breadth is calculated as the percentage of pseudoviruses neutralized with ID50 > 20. The plasma was defined with broadly neutralizing activity if it could neutralize more than 80% of the pseudoviruses tested, indicating a high prevalence of broadly cross-reactive NAbs activities in this plasma [23].

This study was reviewed and approved by the Institutional Review Boards of the National Center for AIDS/STD Control and Prevention, Chinese Center for Disease Control and Prevention, and the subject provided written informed consent prior to blood and data collection.

### 2.2. Viral RNA Extraction, cDNA Synthesis, and Single-Genome Amplification

Viral RNA was extracted from the plasma samples using a QIAamp Viral RNA Mini Kit (Qiagen, Hilden, Germany), and the first-strand cDNA was immediately synthesized by SuperScript First-Strand Synthesis System (Invitrogen, Carlsbad, CA, USA) according to the manufacturer’s instructions. After regular PCR positive identification, PCR amplification was conducted through the single-genome amplification (SGA) method with PrimeSTAR HS DNA Polymerase (Takara, Kyoto, Japan) [24]. The synthesized cDNA was endpoint diluted in 96-well plates such that fewer than 29 PCRs yielded an amplification product. According to a Poisson distribution, the cDNA dilution that yields PCR products in no more than 30% of wells contains one amplifiable cDNA template per positive PCR more than 80% of the time. Based on this theory, cDNA was diluted, and SGA was conducted. The first-round primers were BOB (TAGAGCCTTGGAAGCATCCAGGAAGTCAG (HXB2 5853–5881)) and BOE (TAGCCCTTCCAGTCCCCCCTTTTCTTTTA (HXB2 9096–9068)). The second primers were BIB (CACCGATCAAGCTTTAGGCATCTCCTATGGCAGGAAGAAG (HXB2 5853–5881)) and BIE (AGCTGGATCCGTCTCGAGATACTGCTCCCACCC (HXB2 9096–9068)). The same proportion reaction mixture was used for the first and second rounds of PCR, i.e., a total of 25 μL reaction containing 1 μL of the template with 12.5 μL of 2 × PrimeSART GC buffer, 2 μL of 2.5 mM dNTP mixture, 0.5 μL 5′ primer (10 μM), 0.5 μL 3′ primer (10 μM), 0.25 μL PrimeSTAR HS DNA Polymerase, and 8.5 μL of H2O. The same PCR conditions were used for the first and second rounds of PCR, i.e., 98 °C for 2 min, followed by 30 cycles of 98 °C for 10 s, 55 °C for 5 s, and 72 °C for 2 min, with a final extension at 72 °C for 10 min.

### 2.3. DNA Sequencing, Alignment, and Phylogenetic Analyses

PCR products were sequenced on an ABI 3730 Sequencer after gel purification using a QIAquick Gel Extraction Kit (Qiagen, Hilden, Germany). The full-length envelope sequences were assembled and edited using Sequencher 4.9 (Genecodes, Ann Arbor, MI, USA). Sequences were aligned using GeneCutter (http://www.hiv.lanl.gov/content/sequence/GENE_CUTTER/cutter.html) and BioEdit Sequence Alignment Editor and edited by hand when necessary. Phylogenetic analysis was conducted at the webpage https://embnet.vital-it.ch/raxml-bb/.

### 2.4. Amino Acid Matches/Mismatches Analyses

Highlighter analysis enables to find the amino acids in a query sequence that match or do not match those in a single master sequence. First, a consensus amino acid sequence, as the master sequence, was made at https://www.hiv.lanl.gov/content/sequence/CONSENSUS/consensus.html. The sequence order was adjusted according to the evolutionary tree. A Highlighter plot was drawn at https://www.hiv.lanl.gov/content/sequence/HIGHLIGHT/highlighter_top.html. The amino acids that do not match with the master were assigned a color.

### 2.5. Recombination Analyses

Recombination patterns were evaluated using SimPlot software (version 3.5.1), which was created to plot similarity versus position. Briefly, SimPlot calculates the percent identity of the query sequence to reference sequences and draws a graph in a sliding window, which is moved across the alignment in steps [25]. The intervals for recombination breakpoints based on HXB2 numbering were obtained from Recombination Inference Program (RIP) (https://www.hiv.lanl.gov/content/sequence/RIP/RIP.html). The sequences were segmented according to the recombination breakpoints. Subregion neighbor-joining trees were constructed using MEGA 6.06. Each node was assessed by bootstrap analyses with 1000 replicates, and only significant bootstrap values of 70% or greater were shown at the corresponding nodes.

### 2.6. Variable Loop Length and PNGS Analyses and Prediction of Coreceptor Usage

The variable loop length was determined using the online tool Variable Region Characteristics (https://www.hiv.lanl.gov/content/sequence/VAR_REG_CHAR/index.html) for the V1, V2, V3, V4, and V5 loops. The number of potential N-linked glycosylation sites (PNGSs) was identified using the N-Glycosite at the Los Alamos HIV database website (http://www.hiv.lanl.gov/content/sequence/GLYCOSITE/glycosite.html). Then, the variable loop length and the number of PNGSs of the V1, V2, V3, V4, and V5 loops were compared between different clusters and different time points. Sequence logos were drawn in Weblogo (http://weblogo.berkeley.edu/logo.cgi). The viral tropism prediction was determined according to the amino acid composition of the V3 loop using the Geno2pheno tool (http://coreceptor.geno2pheno.org/index. php) with a false-positivity rate (FPR) cutoff of 20% [26]. The tetrapeptide motifs in the V3 loop were identified through manual searching in the alignment.

### 2.7. Cells

For the study, 293T/17 cells were purchased from the American Type Culture Collection (ATCC) and were used to produce pseudoviruses. TZM-bl cells (also called JC53-BL) were obtained from the National Institutes of Health (NIH) AIDS Research and Reference Reagent Program, as contributed by John Kappes and Xiaoyun Wu. TZM-bl cell is a genetically engineered HeLa cell line expressing CD4, CCR5, and CXCR4, and contains Tat-responsive reporter genes for firefly luciferase and Escherichia coli β-galactosidase under control of the HIV-1 long-terminal repeat [27]. TZM-bl cells were used to test the neutralizing activity of plasma and mAbs. Both cell lines were maintained in Dulbecco’s Modified Eagle’s medium containing 10% heat-inactivated fetal bovine serum and 50 μg/mL gentamicin.

### 2.8. Pseudovirus Preparation and Titration

The strategy to construct and generate pseudoviruses was previously described [28]. Briefly, the env SGA PCR products were cloned into the vector pcDNA 3.1D/V5-His-TOPO (Invitrogen), which allows the env gene to be inserted in the correct orientation with a cytomegalovirus promoter for protein expression. Then, the Env/Rev expression plasmid and Env-deficient HIV-1 backbone vector (pSG3ΔEnv) were transfected into 293T/17 cells using the transfection reagent Polyethylenimine (PEI) (Sigma, St. Louis, MO, USA). At 48 h post-transfection, the supernatant containing pseudovirus was harvested and filtered (0.45 μm pore size), and single-use aliquots (1 mL) were stored at −80 °C.

The optimal tissue culture infectious dose (TCID) of pseudovirus was determined using TZM-bl cells. Serial dilutions of pseudovirus were performed horizontally in quadruplicate wells of 96-well culture plates for a total of 11 dilution steps. Cell control was set at the last column of the 96-well plate. TZM-bl cells containing 75 μg/mL DEAE Dextran were added to each well, and the plate was incubated. After 48 h, 150 μL supernatant was removed from each well, and 100 µL of Ultra-High Sensitivity Luminescence Reporter Gene Assay System (PerkinElmer, Waltham, MA, USA) was added. After cell lysis, the lysate was transferred to a black 96-well plate for luminescence measurement. Wells producing over triple relative luminescence units (RLUs) than cell control were scored as positive [29].

### 2.9. Neutralization Assays

The neutralization sensitivity of pseudoviruses to plasma and bNAbs was analyzed as previously described [29]. All plasma samples were heat inactivated at 56 °C for 1 h before testing. Plasma samples or bNAbs were serially diluted threefold and incubated with 200 TCID50 of pseudovirus for 1 h at 37 °C. TZM-bl cells were added at 1 × 10^4^ cells/well to a plate in DMEM (Hyclone, Logan, UT, USA) growth medium/10% FBS (Hyclone, Logan, UT, USA) containing 11 μg/mL DEAE-dextran (Sigma, St. Louis, MO, USA). Cell controls and virus controls were set. The plates were then incubated at 37 °C 5% CO_2_ for 48 h. Then, 150 μL of the supernatant was removed, and 100 μL of Ultra-High Sensitivity Luminescence Reporter Gene Assay System (PerkinElmer, Waltham, MA, USA) was added to each well. After 2 min of lysis, 150 μL of the cell lysate was transferred to a 96-well black plate, and luminescence was measured using a Victor 3 luminometer (PerkinElmer, Waltham, MA, USA). All neutralization assays were performed in duplicate. The 50% inhibitory dose (ID50) was defined as either the plasma dilution or sample concentration that caused a 50% reduction in relative luminescence units (RLUs), compared to the level of the virus control subtracted from that of the cell control.

### 2.10. Statistical Analyses

GraphPad Prism version 5.0 (GraphPad Software, San Diego, CA, USA) was used to perform the statistical analyses based on *t*-tests. *p*-values ≤ 0.05 were considered significant.

## 3. Results

### 3.1. Phylogenetic Analyses of the Env Sequences

**Figure 1:**
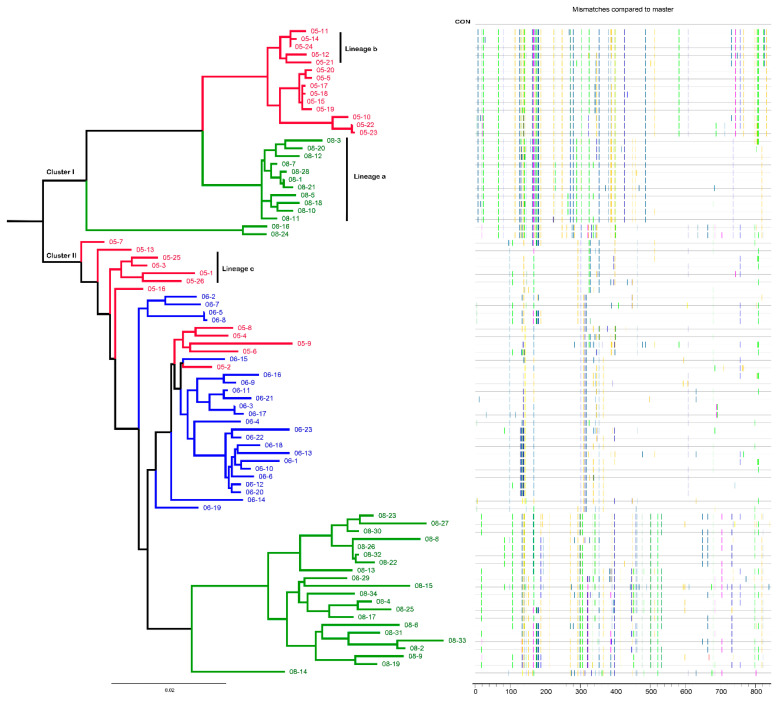
Phylogenetic tree and Highlighter plot of env sequences of CBJC515 from three time points. In the phylogenetic tree, sequences from different sampling time points are highlighted in red (2005), blue (2006), and green (2008), respectively. In the highlighter plot, each line represents an env sequence. The mutations differing from the consensus sequence are color coded according to the amino acid. Lineage a represents a subcluster of 2008 env sequences in Cluster I, lineage b represents a subcluster of 2005 env sequences in Cluster I, and lineage c represents a subcluster of 2005 env sequences in Cluster II. The bar under the phylogenetic tree is the gene distance scale. The scale length (range: 0–1) represents the difference between sequences (range: 0–100%). The x-axis of the highlighter plot indicates the position of the amino acid in gp160.

A total of 83 full-length env sequences were obtained from plasma RNA at three time points (Table 1). A maximum-likelihood tree was constructed to examine the relationship of these env sequences. The phylogenetic tree showed that the 83 env sequences formed two distinct clusters (Cluster I and II) (Figure 1). Cluster I includes 14 sequences from 2005 and 13 sequences from 2008 but does not include sequences from 2006. Cluster II includes 12 sequences from 2005, 23 sequences from 2006, and 21 sequences from 2008. The disappearance of Cluster I strains in 2006 indicated that these viruses may not be adaptive to the host immune environment, and the Cluster I viruses that appeared in 2008 may have new characteristics.

However, a limitation that needs to be considered here is that although a relatively high number of full-length env sequences were obtained in this study, an even much higher number would be needed to exclude that Cluster I virus was present in 2006. However, the 83 env sequences are representative, and the lack of 2006 Cluster I virus in these 83 env sequences at least implies that Cluster I virus present in 2006 is the relatively low-frequency strains that may still represent the disadvantageous characteristics for viral fitness.

### 3.2. Determination of Recombinant Patterns and Breakpoints

The sequence order was adjusted according to the evolutionary tree, and a Highlighter plot was constructed, which is a particularly useful tool for visualizing potential recombination and hypermutation in closely related sequences [30]. From the Highlighter plot, we could observe that the 5′ half of env sequences of lineage a was more similar to the 5′ half of sequences of lineage b, while the 3′ half of env sequences of lineage a was more similar to that of lineage c sequences (Figure 1). This result indicated that viruses of lineage a may be recombinants. Then, we used three different methods to verify whether lineage viruses were recombinants coming from lineage b and lineage c.

**Figure 2:**
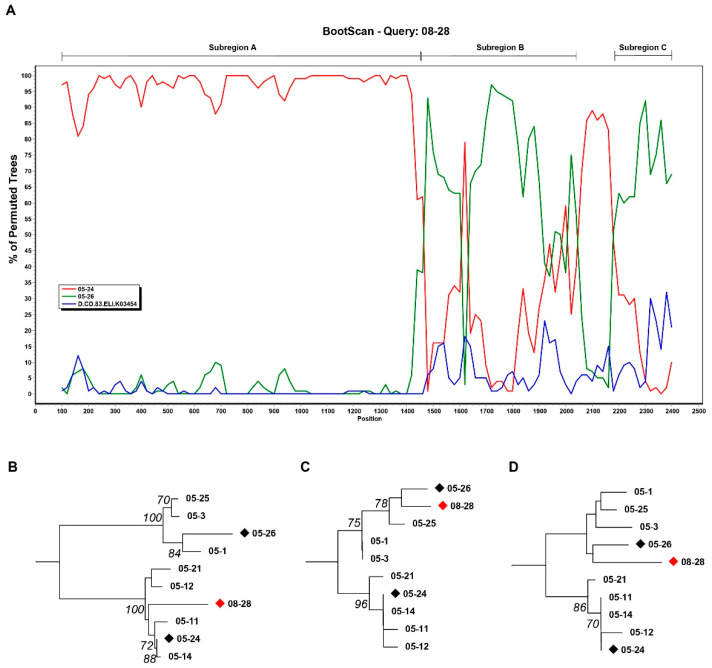
Recombination analysis. (**A**) The similarity between 08-28 and the reference sequence (05-24, 05-26, and D.CD.83ELI.K03454) was plotted using Simplot 3.5.1 software. The x-axis is the nucleotide position in the HIV-1 envelope genomic sequence. The y-axis shows the percentage supporting the clustering with different reference sequences. Subregion A, Subregion B, and Subregion C were indicated. (**B**) The neighbor-joining tree of Subregion A. Sequence 08-28 (nt 6427~7812) clustered with lineage b. (**C**) The neighbor-joining tree of Subregion B. Sequence 08-28 (nt 7816~8282) clustered with lineage c. (**D**) The neighbor-joining tree of Subregion C. Sequence 08-28 (nt 8333~8595) clustered with lineage c.

We randomly selected three viruses, 08-28, 05-24, and 05-26, as representative strains of lineage a, lineage b, and lineage c, respectively. Bootscanning and similarity plot analyses were performed using SimPlot software. SimPlot analysis results showed that the envelope gene of 08-28 was composed of parts of 05-24 and 05-26 (Figure 2A). The recombinant breakpoints were further identified on the RIP website. RIP results revealed that 08-28 was a recombinant originating from 05-24 and 05-26. Alignment with the best match showed that 08-28 (HXB2, 6427~7812) best matched 05-24, while both 08-28 (HXB2, 7816~8282) and 08-28 (HXB2, 8333~8595) best matched 05-26 (data not shown). We named these three 08-28 parts Subregion A, Subregion B, and Subregion C, respectively. Region B and Region C both fell in the gp41 region.

The breakpoint confirmation and each segment origin were analyzed by subregion phylogenetic trees. The subregion neighbor-joining tree showed that Subregion A (Figure 2B) belonged to lineage b and that Subregion B (Figure 2C) and Subregion C (Figure 2D) belonged to lineage c. The bootstrap values of the trees of Subregion A (Figure 2B) and Subregion B (Figure 2C) were both greater than 0.7. The major reason for the reduced confidence (under 0.7) in the tree of Subregion C (Figure 2D) is likely their short sequence length. These results suggested that lineage a might be recombinants and that the parental origin of lineage a sequence was probably lineage b and lineage c. Therefore, we can conclude that the viruses of 2005 Cluster I disappeared in 2006 and then appeared as recombinant strains in 2008, and the breakpoints fell in the gp41 region, indicating that gp41 of Cluster II viruses may retain certain features in Cluster I viruses.

### 3.3. Characteristics of the Variable Loops

**Figure 3:**
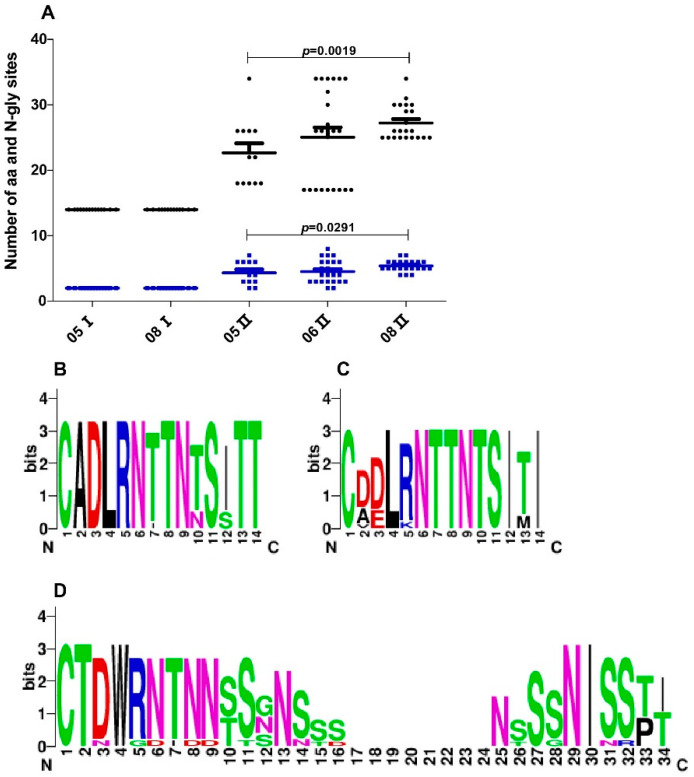
Comparison of sequence length and potential N-linked glycosylation sites (PNGSs) number of the V1 loop. (**A**) Longitudinal analysis of changes in the sequence length and the PNGSs number of Cluster I and Cluster II viruses. Each dot represents one virus variant. Black dots represent V1 loop length. Blue dots represent the V1 loop PNGSs number. The horizontal bars indicate average values per time point, and *p*-values were calculated using a nonparametric t-test for independent samples. (**B**) Sequence logo of V1 of 2005 Cluster I viruses, which depicts the amino acid conservation pattern across a multiple sequence alignment. The height of each letter indicates the degree of conservation of the most common amino acid at that position. (**C**) Sequence logo of V1 of 2008 Cluster I viruses. (**D**) Sequence logo of V1 of 2005 Cluster II viruses.

To try to understand the molecular mechanism contributing to the negative selection of 2006 Cluster I viruses and the persistent existence of Cluster II viruses, we compared the envelope sequence characteristics of the two clusters of viruses. It has been reported that HIV-1 may increase the variable loop length and the number of potential N-linked glycosylation sites (PNGSs) to escape neutralizing pressure by altering or shielding the antibody epitope [31,32]. The loop length and the number of PNGSs of the variable loop, including V1, V2, V3, V4, and V5, were analyzed between the two clusters at three time points. The differences in loop length and number of PNGSs between the two clusters were mainly in the V1 loop (Figure 3A). First, the length and the number of PNGSs of the V1 loop in 2005 Cluster II were both larger than those of 2005 Cluster I. The V1 loop length of 2005 Cluster I and 2005 Cluster II were 14 ± 0 and 22.67 ± 5.07, respectively. The number of PNGSs of the V1 loop of 2005 Cluster I and 2005 Cluster II were 2 ± 0 and 4.33 ± 1.78, respectively. Second, the length and the number of PNGSs of the V1 loop tended to increase with time in Cluster II. The length of the V1 loop in 2008 was significantly longer than that in 2005 (*p* = 0.0019), and the number of PNGSs of the V1 loop in 2008 was more than that in 2005 (*p* = 0.0291) in Cluster II. Third, in Cluster I, the V1 loop retained 14 amino acids and 2 glycosylation sites and was more conserved than that in Cluster II (Figure 3B,D). The lengths of the V2, V3, V4, and V5 loops were 38.10 ± 0.31, 34.99 ± 0.11, 29.53 ± 0.99, and 10.20 ± 0.87, respectively. The numbers of PNGS in the V2, V3, V4, and V5 loops were 2 ± 0, 1 ± 0, 4.41 ± 0.77, and 1.89 ± 0.31, respectively.

Coreceptors, such as CCR5 or CXCR4, are important for HIV-1 virus entry into host cells. HIV-1 infection is almost always established with CCR5-tropic viruses, which predominate during the acute and asymptomatic phases of infection [27]. The appearance of CXCR4-tropic viruses is associated with the progression to AIDS [33]. We predicted the coreceptor usage on the Geno2pheno coreceptor website by offering V3 loop sequences. In Cluster I, all viruses maintained CCR5 tropism. In Cluster II, viruses maintained CCR5 tropism in 2005 and 2006, but 90.5% of 2008 viruses changed to CXCR4 tropism, which may be an indicator of the progression of the disease.

In addition, the GDIR motif around the base of the V3 loop is an important epitope that interacts with long HCDR3 of a series of bNAbs targeting Man8/Man9 glycans in a dense region of the glycan shield [34]. All Cluster I viruses had the amino acid sequence GDIR in the V3 loop. In Cluster II, 51.8% of viruses had GDIR motifs, and the others carried GNIR motifs.

### 3.4. Virus Neutralization Sensitivity to Autologous Plasma

To try to understand the phenotypic property contributing to the negative selection of 2006 Cluster I viruses and the persistent existence of Cluster II viruses, we analyzed the virus neutralization sensitivity to autologous plasma. Virus neutralization sensitivity was measured as a 50% inhibitory dose (ID50) in dilution of the plasma. In HIV-1 infectors, viruses induce the production of NAbs. In turn, NAbs will pressure the virus and promote virus evolution. Viral strains that are sensitive to NAbs are subjected to negative selection, and the escaped strains (antibody-resistant strains) persist and are subject to another round of coevolving and co-selecting with Nabs [19,20,21].

**Figure 4:**
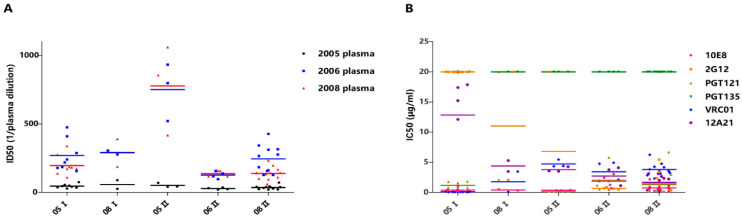
Viruses’ neutralization sensitivity to autologous plasma and bNAbs. (**A**) Comparison of neutralization sensitivity of Cluster I and Cluster II viruses to autologous plasma of three time points (20050816, 20060418, and 20081118). The neutralization sensitivity was measured as ID50 in dilution of the plasma. ID50 < 20 has been set to ID50 = 20. (**B**) Comparison of neutralization sensitivity of Cluster I and Cluster II viruses to several well-known bNAbs. IC50 > 20 μg/mL has been set to IC50 = 20 μg/mL.

A total of 28 infectious pseudotyped viruses were constructed in the current study, 10 of which belong to Cluster I and 18 belong to Cluster II. Furthermore, 08-28 was the only recombinant-pseudotyped virus belonging to Cluster I. The ID50 values of 2005 plasma against 2005 Cluster I viruses and 2005 Cluster II viruses were 45.63 ± 14.66 and 51.33 ± 16.20, respectively. The ID50 values of the 2006 plasma against 2005 Cluster I viruses and 2005 Cluster II viruses were 268.88 ± 115.75 and 750.33 ± 209.93, respectively. The ID50 of 2005 plasma against 08-28 was 89, and the ID50 of 2006 plasma against 08-28 was 276 (Table 3). These results illustrate that 2005 Cluster I viruses, which lacked direct progeny at later stages of infection, did not show more sensitivity than 2005 Cluster II viruses to plasma from 2005 and 2006. Virus 08-28, a recombinant-pseudotyped virus and adaptive strain, did not show more resistance to autologous plasma than 2005 Cluster I viruses (Figure 4). In addition, nearly all pseudoviruses were sensitive to autologous plasma at the three time points except 06-20 and 08-23; these two viruses were resistant to 2005 plasma (Table 3). This result was not in accordance with previous reports that proved that autologous plasma could neutralize HIV-1 viruses from earlier time points but could not neutralize concurrent viruses and later-time-point viruses [35,36,37,38]. This may be why we did not find direct phenotypic evidence that viruses of Cluster I disappeared in 2006, while viruses of Cluster II persisted in this four-year period.

### 3.5. Virus Neutralization Sensitivity to Several Well-Known bNAbs

To determine which kind of bNAbs, similar to representative bNAbs, may exist in this sample to provide clues for the identification and isolation of new bNAbs, we detected pseudovirus neutralization sensitivity to the following monoclonal antibodies: 10E8, 2G12, PGT121, PGT135, VRC01, and 12A21. All viruses were sensitive to 10E8 and VRC01, with average IC50 values of 0.73 ± 0.70 μg/mL and 2.64 ± 1.97 μg/mL, respectively, while all viruses were resistant to the monoclonal antibody PGT135. In addition, 96.4% of the viruses were sensitive to PGT121, and 92.9% of the viruses were sensitive to 12A21. The neutralization sensitivity to 2G12 was significantly different between the two clusters of viruses. Overall, 10% of Cluster I viruses were sensitive to 2G12, while 94.4% of Cluster II viruses were sensitive to 2G12 (Table 2).

In addition, we compared the virus neutralization sensitivity to bNAbs between different clusters and different years. Viruses of 2005 Cluster I were more sensitive to VRC01 than 2005 Cluster II (*p* = 0.006) but more resistant to PGT121 than 2005 Cluster II (*p* = 0.001). This may imply the difference of antibody epitopes between Cluster I and Cluster II in 2005. In Cluster II, the virus sensitivity to 12A21 was gradually increased over time; viruses of 2008 are more sensitive to 12A21 than in 2005 (*p* = 0.001) (Figure 4B).

## 4. Discussion

Deciphering the virus characteristics and their evolution under pressure from bNAbs may lead to further understanding of the mechanism of interaction between HIV-1 virus and humoral immunity. An agreement has been reached that antibody–virus coevolution drives the maturation of bNAbs. However, whether viruses continue to evolve and what the characteristic of virus evolution is under bNAb pressure require further study.

Here, we studied a subtype B’ HIV-1 infected slow progressor, CBJC515, whose plasma has broadly neutralizing activity. We obtained 83 envelope gene sequences from three time points spanning four years. Phylogenic analysis revealed that CBJC515 was infected with two clusters of viruses (Cluster I and II). Interestingly, Cluster II included sequences from all three time points, while Cluster I included only sequences from 2005 and 2008. What led to the disappearance of the direct progeny of 2005 Cluster I? Did 2005 Cluster II viruses have some more favorable characteristics than 2005 Cluster I? What new characteristics might 2008 Cluster I, as a later adaptive strain, have? Sequence alignment and recombinant analysis indicated that 2008 sequences of Cluster I were recombinants. It has been reported that recombination can help viruses form favorable genetic configurations to facilitate faster adaptation [39], which may be more effective than just the accumulation of site mutations alone.

To understand the mechanisms contributing to the negative selection of 2006 Cluster I, we compared the molecular and phenotypic characteristics of the primitively coexisting HIV-1 viral strains that did (2005 Cluster II) or did not (2005 Cluster I) directly generate progeny virus in plasma. We compared the length and PNGSs number of the variable loop of the two clusters. We found that the V1 loop of 2005 Cluster II viruses is longer and had more PNGSs than the V1 loop of Cluster I viruses. In addition, we found that the V1 loop length of Cluster II viruses increased with the sampling time and that the number of PNGSs of the V1 loop of Cluster II viruses also increased over time. These results indicated that there may be neutralizing immune stress targeting the V1 loop in this infector and that Cluster II viruses may escape through increasing V1 loop length and the number of PNGS to alter or shield the antibody epitope. Additionally, these results implied that the short and conserved V1 loop of Cluster I may be a disadvantage factor for its adaptation.

During the antibody and virus coevolution process, viral strains that are sensitive to the antibodies will be subjected to negative selection, while antibody-resistant HIV-1 strains survive. According to this theory, 2005 Cluster I viruses, as negatively selected strains in 2006, should be more sensitive than 2005 Cluster II viruses to 2005 plasma. Virus 08-28, as an adaptive strain, should be more resistant than the 2005 Cluster I virus against plasma from 2005, 2006, and 2008. In fact, the opposite is true. The 2005 Cluster II viruses were not more resistant than Cluster I viruses. In addition, 08-28 was more sensitive than 2005 Cluster I viruses to 2005 and 2008 plasma and on par with the average of Cluster I viruses to 2006 plasma. This result may be for two reasons. First, the neutralizing antibody response is not the main driving force. Second, the half-life of HIV-1 in plasma is approximately 1.3 h [40]. The negative selection of 2005 Cluster I occurred at some point between 2005 and 2006, but we did not obtain direct data to prove it. In addition, we found that the plasma from 2005 and 2006 neutralized both coexisting and evolving viruses and that 2008 plasma could neutralize contemporaneous viruses. Recent studies have found that this strange phenomenon is present in not CBJC515 alone. Similar phenomena have been observed in two other samples, AIIMS_30 [41] and EB354 [42]. The explanation given by Freund was that the coexistence of sensitive HIV-1 strains with effective neutralizing antibodies against the virus may help control the virus in the HIV-1 infector [42]. AIIMS_30 and EB354 are both HIV-1 elite neutralizers; yet, our subject was a slow progressor, indicating that this phenomenon can also occur in slow progressor. However, the mechanism needs further study.

Broad neutralizers are potential candidates for the isolation of new HIV-1 bNAbs, which may serve as reagents for prevention and treatment and can provide materials for studying bNAbs. It is important to predict the potential antibodies in the donor before antibody sorting. Sather et al. proposed a hypothesis that if bNAbs could neutralize early isolates but not late isolates, this situation might indicate that the infected person had developed antibodies related to these epitopes and that the virus had escaped bNAb neutralizing stress [43]. In the same way, if bNAbs could not neutralize all isolates, this might imply that there were possibly similar bNAbs in the infector already. Based on this theory, we may infer which type of bNAbs would be contained in the infector. In this study, most pseudoviruses were sensitive to 10E8, PGT121, VRC01 and 12A21 at the three time points, indicating that there were no similar bNAbs in the infector. All pseudoviruses were resistant to PGT135, indicating that there might already be similar bNAbs in the infector. Interestingly, most 2005 pseudoviruses were resistant to 2G12, while most pseudoviruses of 2006 and 2008 were sensitive to 2G12. This result indicates that two distinct B cell lineages can act “cooperatively” in driving bNAb development; a cooperative B cell lineage selected a virus escape mutant that was resistant to this lineage, but this mutant enhanced the sensitivity to neutralization by the second bNAb lineage [20].

A limitation of this study is that an even much higher number of genome sequences would be needed to exclude that Cluster I virus was present in 2006, although lack of 2006 Cluster I virus in the obtained 83 env sequences might imply the disadvantageous characteristics for viral fitness.

Overall, our findings illuminated that viruses continue to evolve under bNAb pressure and that two cluster viruses adopt different ways to escape immune stress in an infector. Moreover, we studied the evolutionary mechanism of viruses under the pressure of bNAbs, which deepened our understanding of the relationship between bNAbs and viruses. In addition, we inferred that this HIV-1 infector may contain bNAbs related to the V1 loop and bNAbs similar to PGT135, which may contribute to the isolation of bNAbs from this infector.

## 5. Conclusions

A fraction of HIV-1 infected subjects are able to develop bNAbs during natural infection, although how such antibodies arise and the nature of envelope proteins in shaping these responses remain unclear. A better understanding of the genetic characteristics of envelope sequences in natural infection could inform current vaccine strategies aimed at eliciting bNAbs. Here, we investigated the characteristics of sequential HIV-1 envelope sequences and virus neutralization sensitivity to autologous plasmas and several well-known bNAbs in a plasma donor CBJC515 with broadly neutralizing activity, to identify features that potentially contribute to the immunogenic properties of the envelope proteins. A total of 83 full-length HIV-1 env gene sequences longitudinally from three time points spanning four years formed two evolutionary clusters (Cluster I and II), which evolved in different ways. Cluster I viruses vanished in 2006 and then appeared as recombinants two years later. The shorter and conserved V1 loop of Cluster I viruses may be a disadvantage factor for its adaptation. Cluster II viruses may escape through increasing V1 loop length and the PNGSs number to alter or shield the antibody epitope. In addition, 28 Env-pseudotyped viruses were constructed and their neutralization sensitivity to autologous plasma and several representative bNAbs were measured. Nearly all these viruses were sensitive to concurrent and subsequent autologous plasma, a result not in accordance with previous reports that proved that autologous plasma could neutralize HIV-1 viruses from earlier time points but could not neutralize concurrent viruses and later-time-point viruses; the mechanism of this phenomenon needs further study. These viruses were also sensitive to bNAbs including 10E8, PGT121, VRC01, and 12A21, but they were all resistant to PGT135. Taken together, we demonstrate that viruses continue to evolve even in the presence of bNAbs, and two virus clusters in this donor may adopt different escape mechanisms under the same humoral immune pressure.

## Figures and Tables

**Table 1:** The profile of the study subject CBJC515.

Sampling Date	Viral Load (Copies/mL)	CD4 Load (Cell/μL)	Sequences	Gene Distance	Pseudovirus
16 August 2005	19,700	528	26	0.030 ± 0.002	11
18 April 2006	15,500	536	23	0.012 ± 0.001	4
18 November 2008	125,000	321	34	0.028 ± 0.002	13

**Table 2:** Neutralization activities of CBJC515 plasma against DRVI (Division of Research on Virology and Immunology) panel pseudoviruses (*n* = 23).

Clade	Tier	Pseudovirus	ID50
B	2	QH0692	944
	3	PVO.4	1588
	2	REJO4541	156
	2	AC10.0	1867
	3	TRJO4551	1400
	2	CAAN5342	248
	2	SC422661	1453
	2	RHPA4259	358
C	2	Du422	26
	2	ZM249M	38
	1B	ZM109F	75
	2	CAP45	164
CRF07_BC	2	CH117	41
	2	CH181	958
	3	CH120	1106
	2	CH110	997
A	2	Q461	102
	2	Q769	32
	2	Q259	10
	2	Q842	1145
CRF01_AE	NA	BJMSM2249	79
	NA	BJMSM2316	2508
	NA	BJMSM2498	84
GMT = 256.57 *		Breath = 95.65%	

* GMT: geometric mean ID50 titer.

**Table 3:** Neutralization sensitivity of Cluster I and Cluster II viruses to autologous plasma/broadly neutralizing antibodies (bNAbs) ^#^.

	ID	20050816	20060418	20081118	10E8	2G12	PGT121	PGT135	VRC01	12A21
I	05-24	28	178	138	0.42	>20	1.77	>20	0.24	17.39
	05-21	37	156	109	0.31	>20	1.82	>20	0.09	0.05
	05-20	41	288	185	0.33	>20	1.27	>20	0.11	0.07
	05-17	55	475	338	0.34	>20	0.8	>20	0.09	17.86
	05-18	34	185	172	0.96	>20	0.73	>20	0.06	15.21
	05-19	75	409	275	0.22	>20	0.79	>20	0.15	12.12
	05-22	46	241	182	0.22	>20	1.56	>20	0.06	>20
	05-23	49	219	172	0.26	>20	0.55	>20	0.077	>20
	08-28	89	276	390	0.31	>20	2.1	>20	0.08	5.29
	08-24	26	306	188	0.5	2.04	>20	>20	3.45	3.47
II	05-25	41	797	417	0.39	0.21	0.2	>20	4.33	3.56
	05-26	70	933	1060	0.35	>20	0.2	>20	5.45	4.27
	05-16	43	521	855	0.33	0.2	0.18	>20	4.38	3.5
	06-21	36	96	159	0.68	0.89	5.79	>20	2.11	1.91
	06-23	31	138	157	1.24	0.47	0.45	>20	1.88	1.12
	06-22	24	156	116	2.42	0.67	0.53	>20	4.93	3.76
	06-20	<20	117	116	3.05	0.62	1.19	>20	4.76	4.09
	08-23	<20	127	60	2.17	0.77	0.8	>20	4.18	3.11
	08-27	33	426	140	0.14	0.71	0.7	>20	3.5	3.12
	08-26	33	426	140	0.55	0.34	0.33	>20	3.24	2.22
	08-32	22	184	101	0.5	0.46	0.48	>20	4.25	2.32
	08-22	38	266	144	0.86	0.67	0.2	>20	3.54	2.63
	08-29	37	157	95	0.43	5.42	0.31	>20	4.76	0.44
	08-30	39	276	167	0.88	0.58	0.47	>20	3.09	1.99
	08-34	43	315	233	0.7	0.5	0.19	>20	2.84	0.68
	08-25	27	127	109	0.92	3.37	6.67	>20	6.24	0.36
	08-31	40	343	142	0.28	0.91	1.11	>20	3.81	0.1
	08-33	24	162	133	0.73	2.37	2.97	>20	2.31	1.27

^#^ Note: Virus neutralization sensitivity to plasma was measured as ID50 in dilution of the plasma(1/plasma dilution) or antibody concentration (μg/mL).

## Data Availability

The envelope gene sequences of CBJC515 have been submitted to GenBank and assigned accession no. MF591581-MF591629 and MF591646-MF591679. The data generated and/or analyzed during the current study are available from the corresponding author on reasonable request.
